# ERRγ Is Not Required for Skeletal Development but Is a RUNX2-Dependent Negative Regulator of Postnatal Bone Formation in Male Mice

**DOI:** 10.1371/journal.pone.0109592

**Published:** 2014-10-14

**Authors:** Marco Cardelli, Jane E. Aubin

**Affiliations:** 1 Department of Medical Biophysics, University of Toronto, Toronto, Ontario, Canada; 2 Department of Molecular Genetics, University of Toronto, Toronto, Ontario, Canada; Institut de Génomique Fonctionnelle de Lyon, France

## Abstract

To assess the effects of the orphan nuclear Estrogen receptor-related receptor gamma (ERRγ) deficiency on skeletal development and bone turnover, we utilized an ERRγ global knockout mouse line. While we observed no gross morphological anomalies or difference in skeletal length in newborn mice, by 8 weeks of age *ERR*γ *+/−* males but not females exhibited increased trabecular bone, which was further increased by 14 weeks. The increase in trabecular bone was due to an increase in active osteoblasts on the bone surface, without detectable alterations in osteoclast number or activity. Consistent with the histomorphometric results, we observed an increase in gene expression of the bone formation markers alkaline phosphatase (*Alp*) and bone sialoprotein (*Bsp*) in bone and increase in serum ALP, but no change in the osteoclast regulators receptor activator of NF-κB ligand (RANKL) and osteoprotegerin (OPG) or the resorption marker carboxy-terminal collagen crosslinks (CTX). More colony forming units-alkaline phosphatase and -osteoblast (CFU-ALP, CFU-O respectively) but not CFU-fibroblast (CFU-F) formed in *ERR*γ *+/−* versus *ERR*γ *+/+* stromal cell cultures, suggesting that ERRγ negatively regulates osteoblast differentiation and matrix mineralization but not mesenchymal precursor number. By co-immunoprecipitation experiments, we found that ERRγ and RUNX2 interact in an ERRγ DNA binding domain (DBD)-dependent manner. Treatment of post-confluent differentiating bone marrow stromal cell cultures with *Runx2* antisense oligonucleotides resulted in a reduction of CFU-ALP/CFU-O in *ERR*γ *+/−* but not *ERR*γ *+/+* mice compared to their corresponding sense controls. Our data indicate that ERRγ is not required for skeletal development but is a sex-dependent negative regulator of postnatal bone formation, acting in a RUNX2- and apparently differentiation stage-dependent manner.

## Introduction

The Estrogen receptor-related receptors (ERR) are orphan nuclear receptors comprising three family members: ERRα, ERRβ and ERRγ (NR3B1, NR3B2 and NR3B3 respectively) [1]. They are similar in structure to the classic Estrogen receptors, ERα and ERβ, with high amino acid identity in their DNA binding domain (DBD; e.g., over 60% in human ERRα and ERα), but lower (e.g., less than 35%) identity in the ligand binding domain (LBD); the low sequence identity in the LBD is consistent with the observation that the ERRs do not bind Estrogen [2], [3]. Mouse knockout studies have revealed that ERRα and ERRγ are important regulators of energy metabolism [4]–[7]. ERRγ in particular is a key regulator of mitochondrial genes, and its absence results in perinatal lethality, as a consequence of a failure to transition from carbohydrate dependence to fatty acid oxidation [6].

The role of ERRs in bone formation and turnover is also being investigated. ERRα is expressed in osteoblasts throughout the skeleton and was shown to be a positive regulator of osteoblast proliferation and differentiation *in vitro* [8]. There have been conflicting data published on ERRα activity on bone *in vivo*, with data supporting both a negative regulatory [9], [10] and a positive regulatory [11] role. These latter differences may reflect inherent differences in different knockout mouse strains, differences in activities in female [9], [10] versus male [11] mice, and age-related cell autonomous versus non-autonomous roles in bone cells (osteoblasts, osteoclasts) versus other lineages in the bone environment (e.g., adipocytes) [7].

There are fewer studies addressing the role of ERRγ in bone. It has been reported that an ESRRγ polymorphism is associated with bone mineral density in females of European ancestry [12]. Nuclear receptor gene expression profiling has shown *ERR*γ to be expressed early in the differentiation time course of mouse calvaria cells [13] and bone marrow-derived stromal cells [14] *in vitro*, with a steady decrease in expression as differentiation progresses. *ERRγ* expression was reported to be increased in mouse calvaria cells in culture upon stimulation by bone morphogenetic protein 2 (BMP2) and, through protein interaction with RUNX2, to prevent normal cofactor interaction, resulting in repression of transactivation of its target genes, bone sialoprotein (*Bsp*) and osteocalcin (*Ocn*) [15]. ERRγ was also recently reported to regulate BMP-stimulated osteogenesis *in vitro* through up-regulation of the microRNA miR-433, which targeted the 3′-UTR region of *Runx2*, decreasing its protein expression [16]. However, the endogenous role of ERRγ during early bone development and postnatal bone turnover has not yet been characterized.

We report here that ERRγ is not required for skeletal development but is a sex-dependent negative regulator of postnatal bone formation. Further, we show that ERRγ acts in a RUNX2-and apparently differentiation stage-dependant manner *in vitro*.

## Materials and Methods

### Ethics Statement

All experimental procedures were performed in accordance with protocols approved by the Canadian Council on Animal Care and the University of Toronto Faculty of Medicine and Pharmacy Animal Care Committee.

### Mouse gene nomenclature

We followed the mouse nomenclature guide as stated on the Mouse Genome Informatics web page (http://www.informatics.jax.org/mgihome/nomen/short_gene.shtml). Thus, genes are written with first letter capitalized followed by small letters, all italicized, while proteins are written all capitalized and non-italics.

### Animals

The ERRγ knockout mouse line was obtained from the Mutant Mouse Regional Resource Center (MMRRC). The ERRγ null mouse strain originated from Deltagen (San Carlos, CA) by insertion of a lacZ-neomycin cassette into *ERRγ* such that the endogenous gene promoter drives expression of beta-galactosidase and nucleotides from base 586 to 610 of exon 2 were deleted. DNA was isolated from either yolk sacs (embryonic mice) or tail clips (postnatal mice) and were genotyped using the following primers:


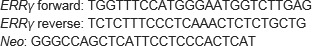


Breeding was performed by crossing heterozygous male with female C57BL/6J mice. For all experiments, littermates were used as controls. Animals were sacrificed by cervical dislocation.

### Whole mount skeletal staining

E15 - P0 animals were dissected, eviscerated and fixed in 95% ethanol overnight or up to two weeks, and then processed for whole mount skeletal staining as previously described [17]. When samples were fully cleared, skeletons were dissected and photographed in a Petri dish containing 100% glycerol, using a Nikon Coolpix P5100 digital camera affixed to a dissecting scope. The images were then quantified in Image J by taking linear measurements of individual skeletal components.

### Microcomputed tomography (µCT)

8, 14, and 52-week old mouse femurs were dissected and stored in 70% ethanol. µCT imaging was performed on a GE Xplore Locus SP imager. A manual trace beginning from just below to 2 mm below the growth plate of the distal femur was used to analyze the cancellous bone. The cortical bone region of interest was defined as a 2 mm long region beginning 2 mm below the growth plate. For P0 mice the whole femur was analyzed. Quantification was performed by an observer blinded to genotype.

### Histology and histomorphometry

14-week old male femurs were dissected and fixed in 4% paraformaldehyde (PFA) overnight at 4°C, dehydrated and stored in 70% ethanol prior to methylmethacrylate (MMA) embedding and sectioning. The distal femur was used to evaluate all histomorphometric parameters. 5 µm sections were double stained for Von Kossa/toluidine blue to evaluate osteoblast properties, or tartrate-resistant acid phosphatase (TRAP)-stained according to manufacture's instructions (387A TRAP kit, Sigma-Alderich) to evaluate osteoclast properties. These measurements were performed on 3 separate sections from each animal, and the average number or surface was calculated. To evaluate dynamic properties, mice were injected intraperitoneally with 30 µg/g body weight of calcein (C0875, Sigma-Alderich) 10 and 3 days prior to dissection. Mineral apposition rate (MAR) was calculated by measuring the distance between 2 calcein labels and dividing by 7 days. Four different regions within the trabecular area were used for calculations. BFR was calculated by multiplication of MAR by the ratio of mineralizing surface (MS) (calcein positive) to the bone surface (BS). Five images were taken at different regions within a section, and 3 different sections were used per animal, and the average was calculated. All quantification was performed by observers blinded to genotype.

### Immunohistochemical detection of Ki67 and TUNEL assay

To immunodetect Ki67, a common proliferation marker [18], sections were de-plasticized, and rehydrated in ethanol washes, followed by antigen retrieval in citrate buffer (10 mM citric acid, 0.05% Tween-20, pH 6) in a 65°C water bath overnight. The slides were blocked in normal goat serum (Invitrogen) for 30 minutes at room temperature, washed, incubated with rabbit polyclonal anti-Ki67 antibody (diluted 1∶25 in blocking buffer) for 1 hour at room temperature, washed, then incubated with biotinylated goat anti-rabbit secondary antibody, and visualized using the Vectastain Elite ABC kit (Vector Labs, Burlingame, California), and counterstained with methylene blue.

To perform TUNEL assay, femoral sections were processed as described above, before using the FragEL DNA fragmentation detection kit (Calbiochem), as per the manufacturers instructions, and counterstained with methyl green. In each case, femoral sections were imaged, and Ki67 (or TUNEL) positive and negative cells on the trabecular bone surface were quantified using Bioquant Osteo 2012; only cells with the morphological characteristics of osteoblasts were quantified. All quantification was performed by observers blinded to genotype.

### Serum biochemistry

Whole blood was collected through the saphenous vein, and the plasma was separated from whole blood by centrifugation and stored at −80°C until biochemical analysis (Vita-Tech, Ontario, Canada). OPG and RANKL in serum were assayed using a Quantikine M Murine OPG ELISA kit and a Quantikine M Murine TRANCE/RANKL ELISA kit (No. MOP00 and No. MTR00, R&D Systems, Minneapolis, MN), respectively following the manufacturer’s directions. CTX-1 levels in serum was determined from fasted mice using Serum CrossLaps ELISA (RatLaps EIA No. AC-06F1, Immunodiagnostic Systems, Fountain Hills, AZ). Serum testosterone levels were quantified by Cornell University Animal Health Diagnostic Center (Ithaca, NY).

### Gene expression analysis

The trabecular bone from 14-week old male femur and tibiae were dissected, had marrow removed, and were manually ground with a mortar and pestle under liquid nitrogen. Stromal cell cultures were rinsed twice with phosphate buffered saline. In either case, total RNA was extracted using TRIzol (Invitrogen), and reverse transcribed using Superscript II Reverse Transcriptase (Invitrogen), according to the manufacturer’s directions. All primers were designed with intron inclusion in corresponding genomic DNA, and are common to all potential transcript variants (Table 1). The reactions were performed in triplicate on a 96-well plate in a BioRad MyIQ iCycler, for 50 cycles with an annealing temperature of 59°C. The amplification data was uploaded into the PCR miner program (http://www.ewindup.info/miner/version2/) to obtain the Ct and reaction efficiency values. The relative expression levels of the target gene were normalized to expression of ribosomal protein *L32* as internal control; we confirmed that the expression of *L32* remained constant throughout cultures under the conditions used, including in knockdown experiments.

**Table 1:** Primer sequences used in gene expression analysis.

Gene	Upstream sequence	Downstream sequence
*L32*	CACAATGTCAAGGAGCTGGAAGT	TCTACAATGGCTTTTCGGTTCT
*Bmp2*	CTCAGCGAATTTGAGTTGAGGC	GGCTTCTAGTTGATGGAACGTG
*Runx2*	TGTTCTCTGATCGCCTCAGTG	CCTGGGATCTGTAATCTGACTCT
*Osx*	ATGGCGTCCTCTCTGCTTG	TGAAAGGTCAGCGTATGGCTT
*Alp*	CCAACTCTTTTGTGCCAGAGA	GGCTACATTGGTGTTGAGCTTTT
*Bsp*	CAGGGAGGCAGTGACTCTTC	AGTGTGGAAAGTGTGGCGTT
*Ocn*	CTGACCTCACAGATCCCAAGC	TGGTCTGATAGCTCGTCACAAG
*Nfatc1*	CAGCTGTTCCTTCAGCCAAT	GGAGGTGATCTCGATTCTCG
*Ctsk*	ACCCATATGTGGGCCAGGATGA	GAGATGGGTCCTACCCGCGC

### Isolation of Bone Marrow Cells, *Runx2* antisense assay, and CFU assay

Bone marrow cells were isolated from dissected tibiae and femora, using a modification of a previously published method [19]. Cells were plated in α-MEM supplemented with 10% heat-inactivated fetal bovine serum (FBS) and antibiotics (1 IU penicillin, 1 µg/mL streptomycin, 50 µg/mL gentamicin, 250 ng/mL fungizone) (standard medium) at 3×10^6^ nucleated cells/35-mm dish. After 4 days, the medium was changed to differentiation medium (standard medium with 50 µg/mL ascorbic acid and 10 mM β-glycerophosphate). For *Runx2* antisense assay, differentiation medium was supplemented with 10 µM *Runx2* oligonucleotides from d5–d19 of culture. *Runx2* oligonucleotide sequences were as follows, as previously described [20]: *Runx2*AS A*G*T*G*TGG TAG TGA GTG GT*G*G*C; *Runx2*S G*C*C*ACC ACT CAC TAC CA*C*A*C (*denotes phosphorothioate modification) (Integrated DNA Technologies, Coralville, Iowa). At day 19, cultures were stained for ALP activity and mineralization (Von Kossa), counted, then re-stained with methylene blue.

### Stable cell line constructs

The plasmids pcDINmERRγ2, pcDINmERRγ2ΔAF2 and pcDINmERRγ2C148G have been described [21]. For the generation of stable overexpressing MC3T3-E1 clone 26 cell lines, 20 µg of plasmid was linearized with SspI and precipitated with ethanol and then air dried. The plasmid pellet was resuspended in 200 µl of α-MEM and then combined with 200 µl α-MEM with 40 µl Lipofectamine 2000 (Invitrogen) before being added onto the cells at 60% confluence in a 10 cm dish. The following day medium was changed to allow the cells to recover. On day two after transfection, regular medium was supplemented with 180 µg/ml (final) of active G418. One non-transfected plate of cells was used as a control. After 2–3 weeks of selection more than 10 colonies could easily be identified on the plate for any of the transfected plasmids and the G418 was reduced to 120 µg/ml until the plate grew confluent. Cells were split for expansion and freezing. When thawing out the vials for experiments, the cells were plated in 180 µg/ml of G418 for initial expansion to ensure high levels of expression, but subsequent passaging was in medium containing 120 µg/ml of G418. High levels of expression were verified by RT-qPCR and Western blot.

### Co-immunoprecipitation Analysis

Co-IP assay was performed essentially as previously reported [15]. Briefly, stable overexpressing MC3T3-E1 cell lines were lysed in Co-IP buffer, and lysates were precleared with 50 µl of pansorbin cells (Calbiochem) for 2 h, which were removed by centrifugation. A total of 2 µg of rabbit polyclonal antibody against RUNX2 (Santa Cruz Biotechnology), or normal rabbit IgG (negative control) were added to the precleared lysates, and incubated at 4°C overnight with rotation. After washing cells in Co-IP buffer, the samples were centrifuged, and SDS sample buffer was added, and boiled. The immunoprecipitated complexes were separated by SDS-PAGE, transferred to polyvinylidene difluoride, and immunoblotted with a rabbit polyclonal antibody against ERRγ (Santa Cruz Biotechnology).

### Western blotting

Whole cell extracts were lysed in RIPA buffer (50 mM Tris HCl pH 8, 150 mM NaCl, 1% NP-40, 0.5% sodium deoxycholate and 0.1% sodium dodecyl sulphate) with added protease inhibitors. Protein samples were quantified using the Bio-Rad DC Protein Assay kit, following the manufacturer’s instructions. Thirty µg of each sample was run in a 10% SDS-PAGE gel, transferred to polyvinylidene fluoride (PVDF) membrane, followed by blocking in 5% milk-TBS-T for 30 minutes at room temperature. Immunodetection was carried out using a rabbit polyclonal anti-ERRγ antibody (H38x, Santa Cruz Biotechnology Inc.) diluted 1∶5000 in blocking buffer, or rabbit polyclonal anti-RUNX2 antibody (Santa Cruz Biotechnology) diluted to 1∶1000, or rabbit anti-β-ACTIN antibody diluted to 1∶2000 (Sigma). This was followed by a one hour incubation with a goat anti-rabbit IgG, conjugated to horse radish peroxidase (HRP; Santa Cruz Biotechnology), diluted 1∶5000–1∶8000 in blocking buffer. The HRP was visualized using WEST-one Western Blot Detection, as per the manufacturer’s instructions. Densitometry was performed using Image Lab software (Bio-Rad). The proteins of interest were normalized to the β-ACTIN band to assess proportionate protein levels.

### Statistical Analysis

All data were analyzed using Graphpad Prism 4.0 software, or Microsoft Excel 2003 software. In most cases, datasets were compared using student's t-Test. When three datasets were analysed, ANOVA was used to determine significance. All the graphs are plotted as the mean ± standard deviation and the p values listed are for the comparison to the WT values. Graphs were constructed using Microsoft Excel 2003 software.

## Results

### *ERRγ −/−* mice die perinatally, but display no skeletal abnormalities

ERRγ null mice (*ERRγ −/−*) died perinatally, with no pups observed beyond P1. This is consistent with the observation that mice of a different *ERRγ −/−* mouse strain, made using a similar gene targeting strategy, also die perinatally due to mitochondrial abnormalities and impaired oxidative metabolism [6]. *ERRγ +/−* mice were healthy and viable, and were used for subsequent breeding.

**Figure 1:**
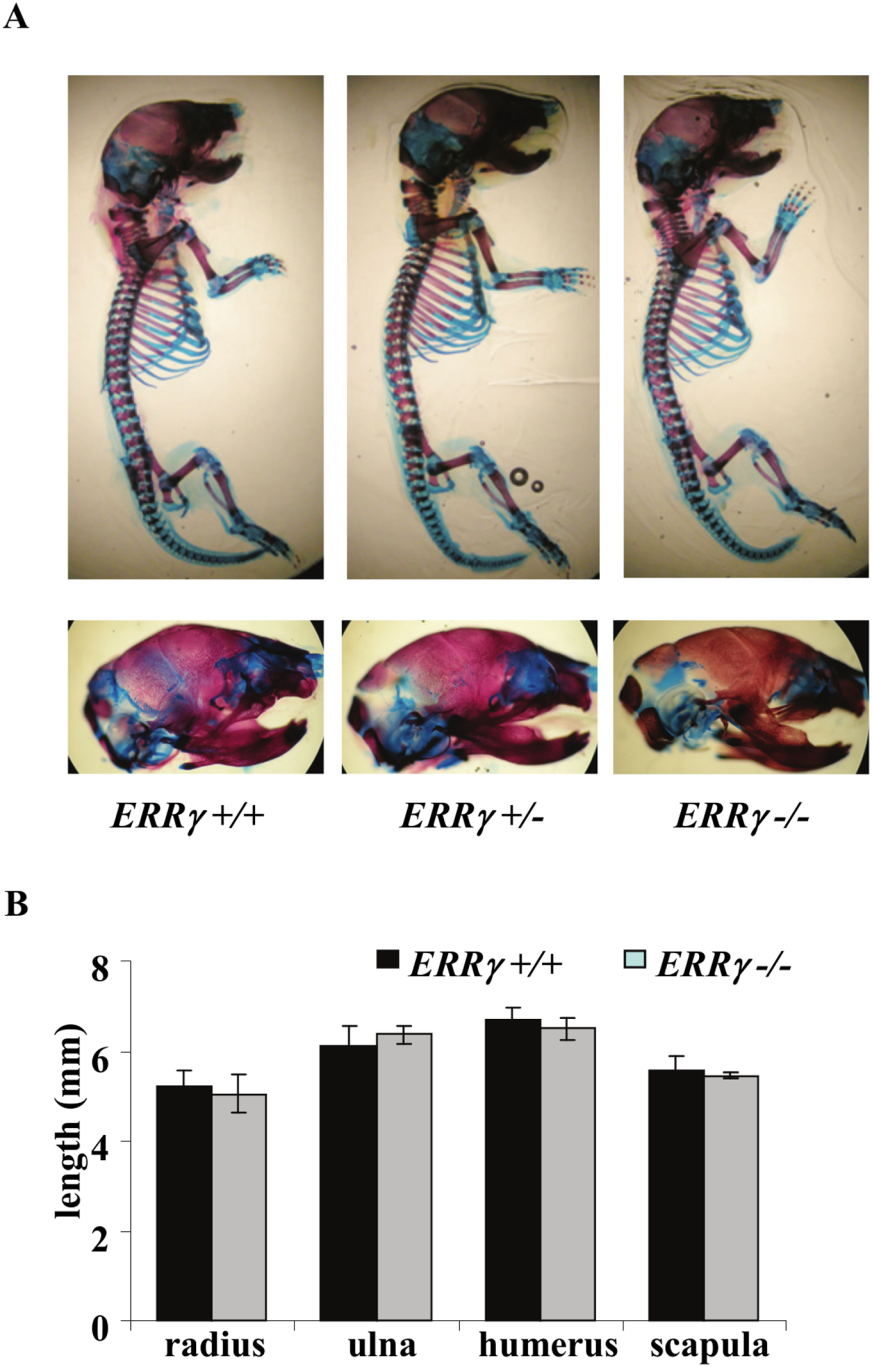
Neither ERRγ ablation nor haploinsufficiency has a detectable effect on embryonic bone development and growth. (A) Whole mount alizarin red/alcian blue staining revealed no observable morphological differences in newborn mouse *ERRγ +/+, ERRγ +/− and ERRγ −/−* skeletons (upper panel) or in the craniofacial regions (lower panel). (B) Skeletal elements that make up the mouse forelimb of each genotype were measured and found to have no significant differences. (N = 5 *ERRγ +/+*; N = 3 *ERRγ −/−*). Values are expressed as mean ± SD.

No significant differences in forelimb length were observed between E15-P0 in *ERRγ +/+, ERRγ +/−* and *ERRγ −/−* whole mount skeletal preparations (Figure 1A, B, P0 shown). There was also no significant difference observed in any of the growth plate zones in sections of P0 humeri (data not shown). MicroCT (µCT) analyses confirmed no significant difference in bone mineral density, content, or bone volume fraction between genotypes in P0 mice (data not shown). Taken together, the data indicate that ERRγ, similarly to ERs and ERRα [9], [10], [22], [23], either plays no apparent role or plays a redundant role in embryonic/early postnatal endochondral bone growth and mineralization.

### Adult *ERRγ +/−* male mice have increased trabecular bone, increased osteoblast number and surface, but no change in osteoclast number or surface

**Figure 2:**
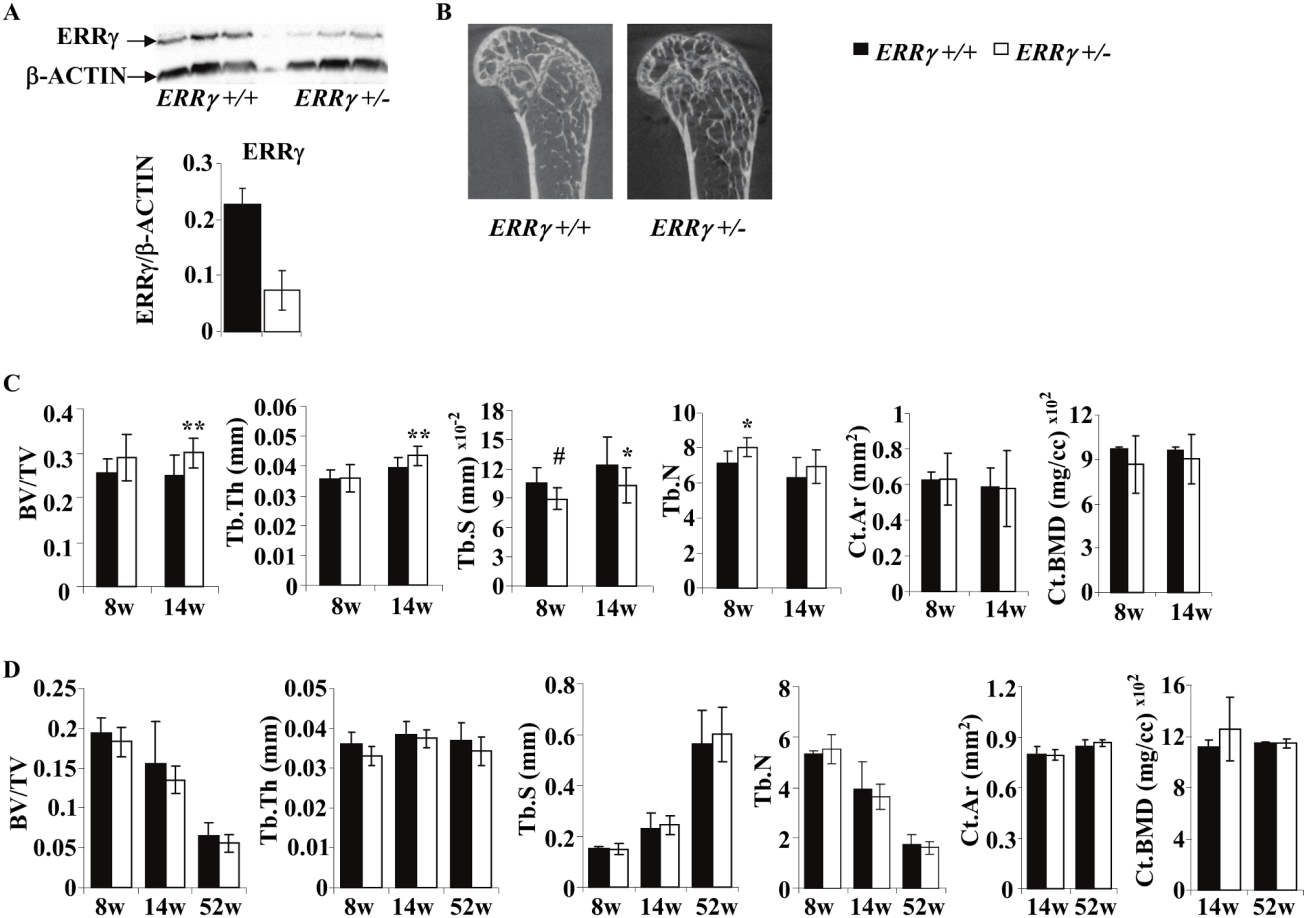
Trabecular bone formation is increased in 14-week old male distal femurs. (A) Quantification of Western blots revealed that ERRγ protein expression was reduced in whole cell lysates of trabecular bones of 14-week old *ERRγ +/−* mice compared to *ERRγ +/+* mice. (B) Representative µCT images of 14-week old *ERRγ +/+* and *ERRγ +/−* male distal femurs. (C) Quantitative analysis revealed a significant increase in trabecular bone volume fraction (BV/TV) and thickness (Tb.Th), and a decrease in separation (Tb.S) at 14 weeks. Trabecular number (Tb.N) was significantly increased at 8 weeks, but not significantly at 14 weeks (upper panels). There were no significant differences in cortical bone area (Ct.Ar) or bone mineral density (BMD) (lower panel). (D) Analysis on 8, 14 and 52-week old female mice revealed no differences in any bone parameters assessed. Values are expressed as mean ± SD (Male: 8w: N = 7 *ERRγ +/+*; N = 5 *ERRγ +/−*; 14w: N = 10 *ERRγ +/+*; N = 14 *ERRγ +/−;* Female: 8w: N = 3 *ERRγ +/+*; N = 5 *ERRγ +/−*; 14w: N = 10 *ERRγ +/+*; N = 7 *ERRγ +/−*; 52w: N = 6 *ERRγ +/+*; N = 5 *ERRγ +/−*) * = p<0.05; ** = p<0.01; # = 0.069.

To address whether ERRγ plays a role in adult bone, we first quantified ERRγ by immunoblotting of whole cell lysates of femoral trabecular bone of 14-week old mice; ERRγ protein was reduced approximately 67% in *ERRγ +/−* mice compared to *ERRγ +/+* (Figure 2A). µCT analyses of 8- and 14-week old male mice (representative image shown in Figure 2B) was performed. Trabecular bone volume (BV/TV) and thickness (Tb.Th) were increased (21% and 10.8%, respectively) at 14 weeks, while trabecular separation (Tb.S) showed a trend (p = 0.069) towards a decrease at 8 weeks, and a significant decrease (16.5%) at 14 weeks in *ERRγ +/−* versus *ERRγ +/+* mice (Figure 2C). Trabecular number (Tb.N) was significantly increased (12.4%) at 8, but not 14 weeks of age. The differences observed were specific to trabecular bone, as there were no changes detectable in cortical bone parameters, including cortical area (Ct.Ar) and cortical BMD (Ct.BMD) (Figure 2C), or when periosteal and endosteal perimeters were quantified (data not shown). Further, no significant differences were observed in any of these parameters in female *ERRγ +/−* mice (Figure 2D).

**Figure 3:**
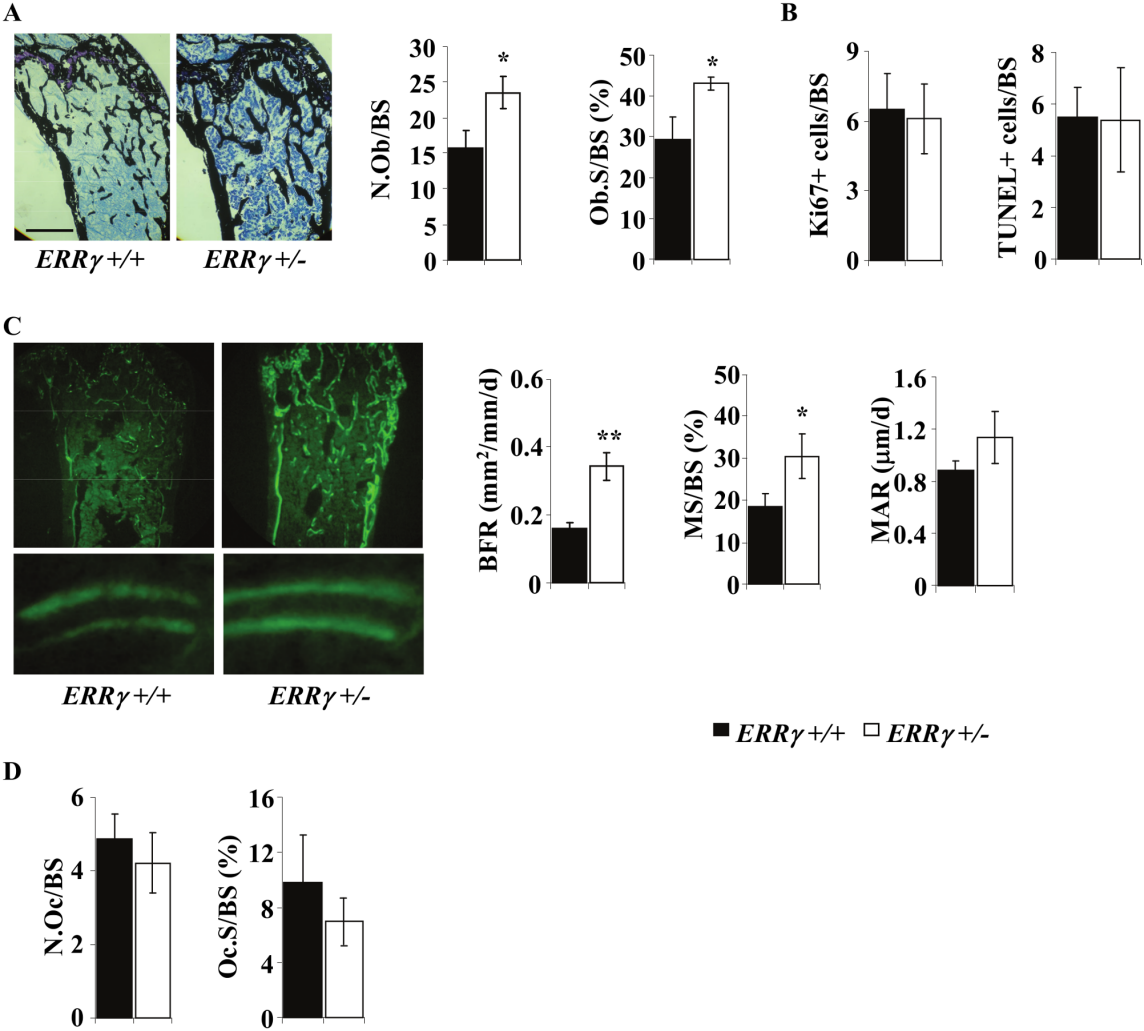
ERRγ regulates osteoblast number and differentiation, but does not affect osteoclast number or activity. (A) von Kossa/toluidine blue double stain was used to quantify osteoblast number and osteoblast surface per bone surface; both are significantly increased in 14 week *ERRγ +/−* compared to *ERRγ +/+* male femurs. Scale = 50 µm (N = 3 *ERRγ +/+*; N = 3 *ERRγ +/−*) (B) Adjacent femoral sections were immunostained for proliferating (Ki67) and apoptotic (TUNEL) osteoblasts; no significant differences were seen between genotypes. (N = 5 *ERRγ +/+*; N = 4 *ERRγ +/−*) (C) Double calcein labels were used to quantify bone formation rate (BFR), mineralizing surface per bone surface (MS/BS) and mineral apposition rate (MAR); both BFR and MS/BS were significantly increased, while MAR was not significantly increased in *ERRγ +/−* compared to *ERRγ +/+* mice. (N = 3 *ERRγ +/+*; N = 3 *ERRγ +/−*) (D) There was no significant difference observed between genotypes in either TRAP-positive osteoclast number (Oc.N/BS) or osteoclast surface per bone surface (Oc.S/BS) (N = 5 *ERRγ +/+*; N = 4 *ERRγ +/−*). All values are expressed as mean ± SD. * = p<0.05; ** = p<0.01.

Histomorphometric analyses indicated that osteoblast number per bone surface (N.Ob/BS) and osteoblast surface per bone surface (Ob.S/BS) were increased in 14-week *ERRγ +/−* compared to *ERRγ +/+* male femurs (Figure 3A). Ki67, an established marker of proliferating cells, and TUNEL staining indicated that neither changes in proliferation nor in apoptosis could account for this increase (Figure 3B). Consistent with the increased N.Ob/BS and Ob.S/BS, bone formation rate (BFR) and mineralizing surface per bone surface (MS/BS) were significantly increased, with no significant difference in mineral apposition rate (MAR) (Figure 3C). No significant difference was seen in either osteoclast number per bone surface (N.Oc/BS) or osteoclast surface per bone surface (Oc.S/BS) in *ERRγ +/−* versus *ERRγ +/+* trabecular bone (Figure 3D).

**Figure 4:**
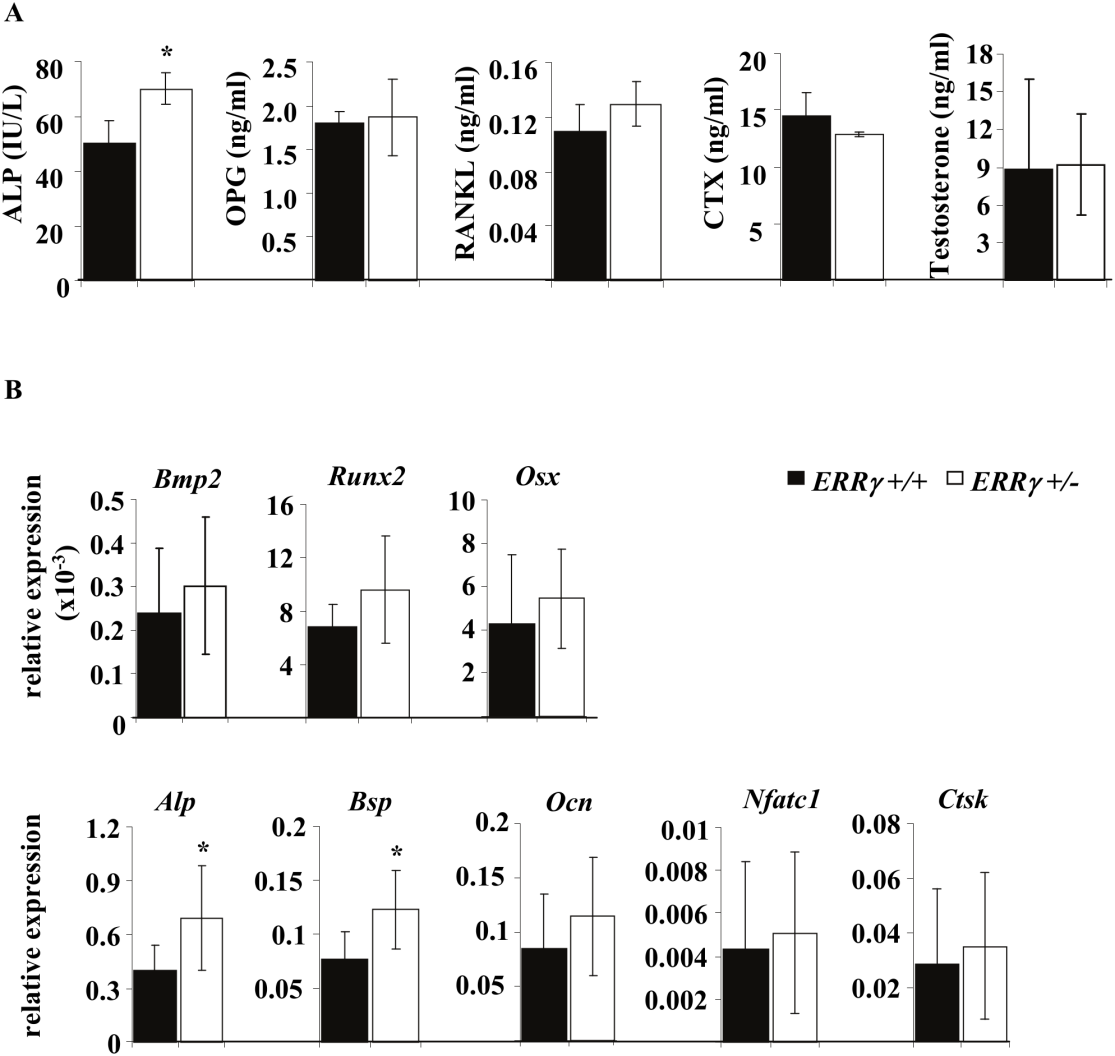
ERRγ regulates markers of osteoblast differentiation and mineralization. (A) Serum analysis of bone turnover markers revealed a significant increase in ALP, an indication of increased bone formation, with no changes in the bone resorption marker CTX or osteoclastogenic factors (OPG, RANKL) or serum testosterone. (N = 4 *ERRγ +/+*; N = 4 *ERRγ +/−*) (B) Gene expression analysis of trabecular bone from 14-week old mice revealed an increase in formation markers and BMP target genes *Alp* and *Bsp*, but no difference in *Bmp2*, *Runx2, Osx, Ocn*, and no difference in the osteoclast markers *Nfatc1* and *Ctsk*. (N = 8 *ERRγ +/+*; N = 6 *ERRγ +/−*). All values are expressed as mean ± SD. * = p<0.05.

Consistent with the morphometric analyses, a statistically significant increase in serum alkaline phosphatise (ALP) levels, a marker of bone formation, was observed in *ERRγ +/−* compared to *ERRγ +/+* mice, while no significant differences were seen between genotypes in serum RANKL, OPG or CTX (Figure 4A). Because adult male ERα knockout mice also exhibit high trabecular bone volume and high serum levels of testosterone, which account for the bone phenotype [24], [25], we assessed serum testosterone levels; no significant difference in testosterone levels between genotypes were detectable (Figure 4A).

Taken together, the data indicate that the high trabecular bone phenotype observed in *ERRγ +/−* male mice was due to an increased number of active osteoblasts but no change in osteoclast number, activity (Figure 3D) or osteoclast marker (*Nfatc1, Ctsk*) gene expression (Figure 4B), suggesting that ERRγ regulates bone formation but not bone resorption in male but not female mice.

### Increased osteoblast differentiation in *ERRγ +/−* mice

**Figure 5:**
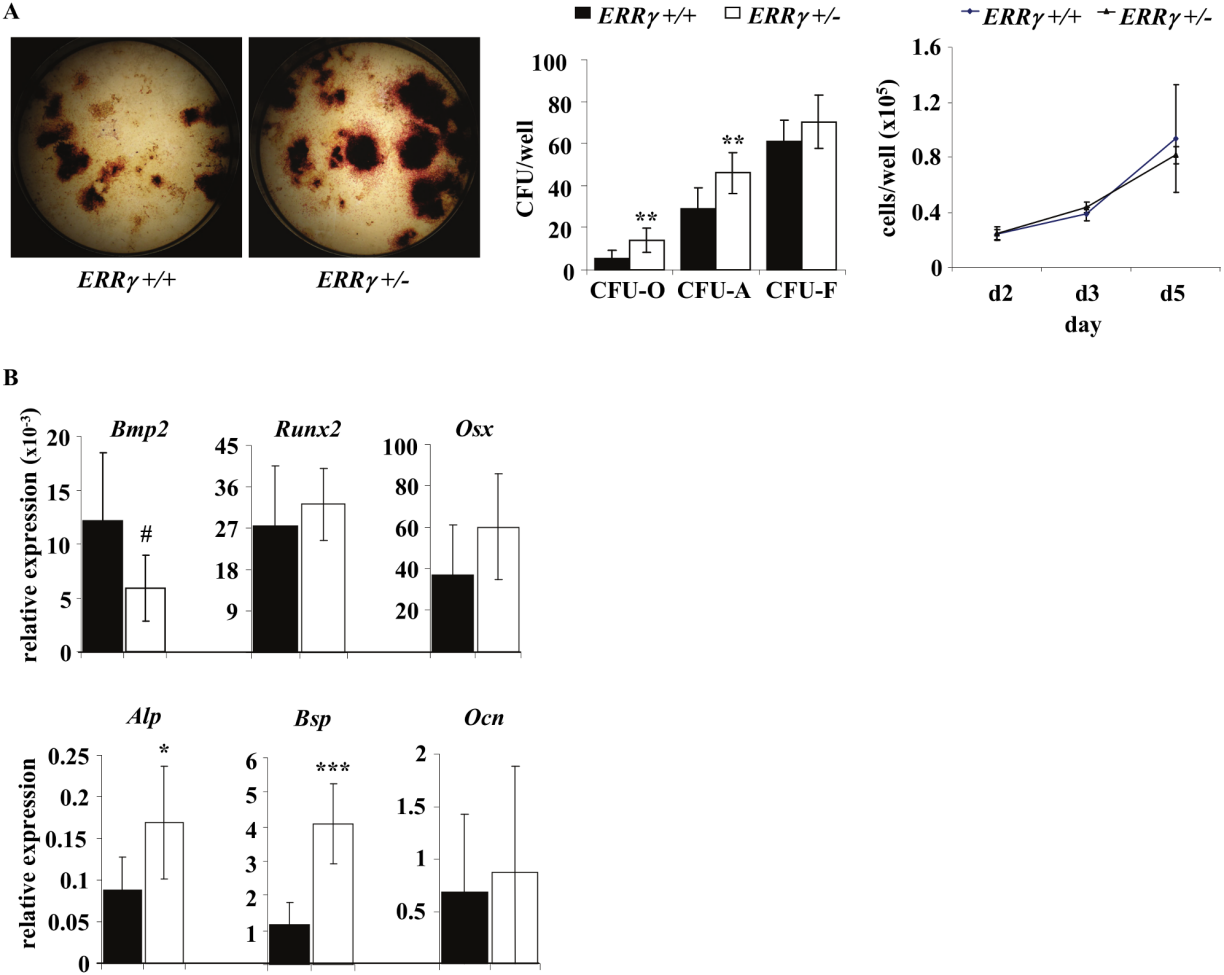
Osteoblast differentiation is increased in *ERRγ* +/− stromal cell cultures. (A) Bone marrow stromal cells cultured from *ERRγ +/−* mice had significant increases in CFU-O and CFU-ALP, with no change in CFU-F, compared to cells from *ERRγ +/+* mice. Cell counts performed during the proliferation phase revealed no differences between genotypes (N = 8 *ERRγ +/+*; N = 8 *ERRγ +/−*). (B) Gene expression analysis of *ERRγ +/−* versus *ERRγ +/+* cultured stromal cells revealed a trend to decreased expression of *Bmp*, and a significant increase in *Alp* and *Bsp* (N = 6 *ERRγ +/+*; N = 6 *ERRγ +/−*). All values are expressed as mean ± SD. # = p = 0.055; * = p<0.05; ** = p<0.01; ***p<0.001.

The data thus far suggest that ERRγ negatively regulates bone formation through regulation of osteoblast number. We therefore next assessed osteoblast differentiation in primary cultures of bone marrow stromal cells from *ERRγ +/−* and *ERRγ +/+* mouse hind limbs. Colony forming unit-osteoblast (CFU-O) and CFU-alkaline phosphatase (CFU-ALP) numbers were increased in *ERRγ +/−* versus *ERRγ +/+* stromal cell cultures, without an increase in CFU-fibroblast (CFU-F) number (Figure 5A). Consistent with the *in vivo* analyses (Figure 3B), no difference was detectable in proliferation of stromal cells between genotypes (Figure 5A).

Because ERRγ has previously been implicated as a negative regulator of BMP-stimulated osteogenesis *in vitro* [15], we next assessed expression of *Bmp* and its downstream targets *in vivo and in vitro*. *Runx2* expression was unchanged in stromal cell cultures (Figure 5B), consistent with the results of Jeong *et al.* [15], or in trabecular bone (Figure 4B) of 14-week old *ERRγ +/−* versus *ERRγ +/+* male mice. *Bmp2* expression was also either unchanged (Figure 4B) or exhibited a downward trend (p = 0.06) (Figure 5B) in *ERRγ +/−* versus *ERRγ +/+* trabecular bone and differentiated stromal cells respectively. Expression of *Alp* and *Bsp* but not *Osx* or *Ocn* was increased in both trabecular bone (Figure 4B) and stromal cells (Figure 5B) of *ERRγ* +/− versus *ERRγ* +/+ mice. There were no significant differences in expression of osteocyte markers (*Dmp1* and *Sost*) or in ERα, ERβ, ERRα or ERRβ in either stromal cell cultures or in trabecular bone samples (data not shown). Taken together, the data suggest that ERRγ negatively regulates osteoblast differentiation and matrix mineralization at a stage after osteoblast commitment (i.e., after upregulation of *Runx2* and *Osx* expression) but before osteoblast maturation or terminal differentiation to osteocytes (i.e., prior to acquisition of *Ocn* expression).

### ERRγ interacts with RUNX2, and knockdown of RUNX2 *in vitro* leads to at least partial rescue of the *ERRγ +/−* osteogenic phenotype

**Figure 6:**
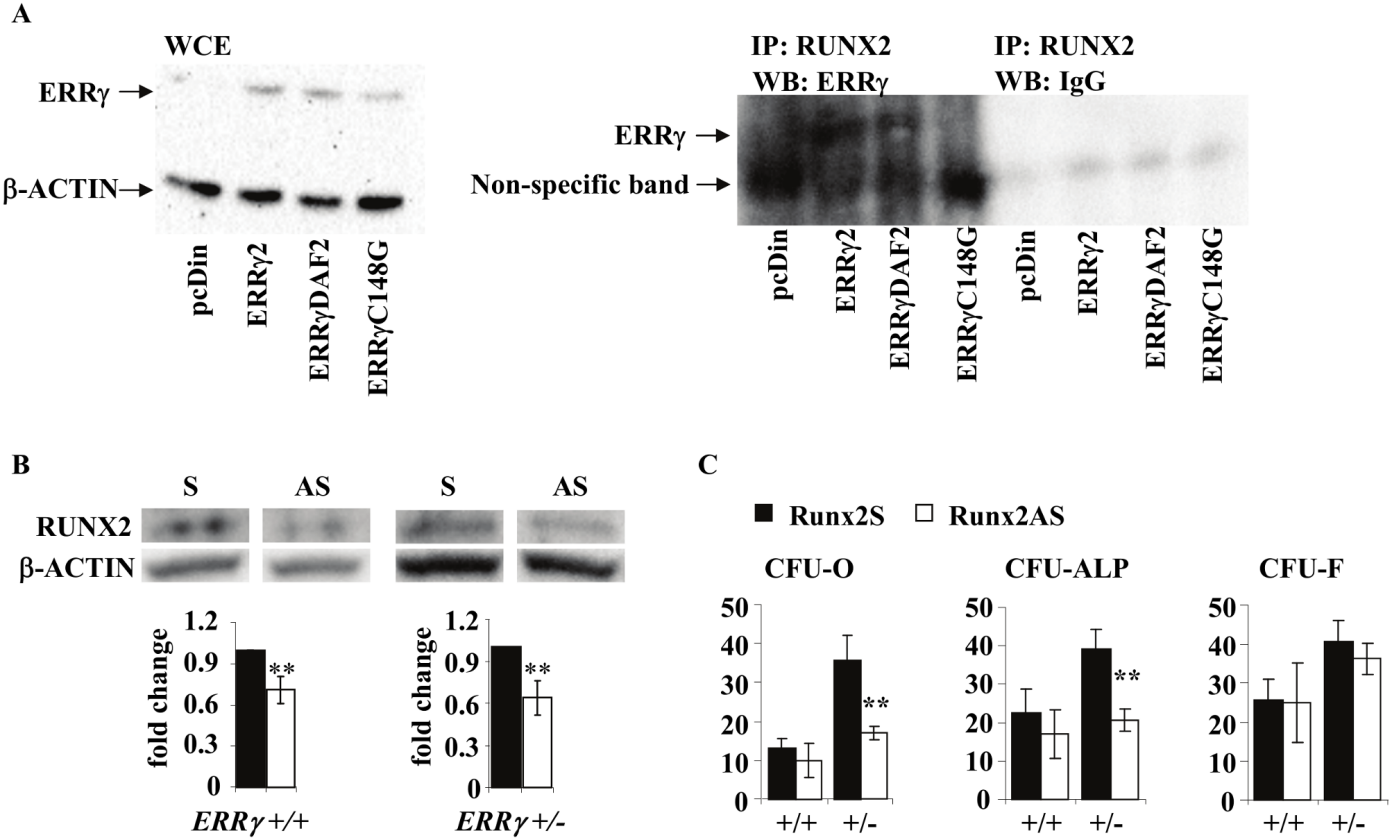
ERRγ interacts with RUNX2 through the DBD and modulates osteoblast differentiation. (A) The MC3T3-E1 preosteoblastic cell line was stably transfected with the indicated plasmids. Whole cell extracts (WCE) from these cell lines were probed with anti-ERRγ, confirming protein expression in the stable constructs. A co-immunoprecipitation assay revealed interaction between RUNX2 and full length ERRγ (ERRγ2), as well as with the LBD mutant form of ERRγ (ERRγΔAF2), but not the ERRγ DBD mutant (ERRγC148G). (B) Decreased protein levels of RUNX2 in *Runx2* antisense (*Runx2*AS), compared to *Runx2* sense (*Runx2*S) samples. (C) CFU-O and CFU-ALP in *ERRγ +/−* bone marrow stromal cells treated with *Runx2* antisense oligonucleotides (*Runx2*AS) were significantly reduced while no changes in either CFU-O or CFU-ALP were observed in *ERRγ +/+* cultures. CFU-F numbers were unchanged regardless of treatment and genotype. All values are expressed as mean ± SD. Graphs shown in (C) are of one representative sample, done in triplicate. In all cases, statistical analyses were performed on a minimum of 3 biological samples. ** = p<0.01.

ERRγ has been reported to outcompete P300 as a co-factor for RUNX2, resulting in repression of RUNX2 transactivity [15]. However, it has not been reported which region(s) of ERRγ is/are required for this interaction, but both the DNA binding domain (DBD) and ligand binding domain (LBD) contain dimerization interfaces that modulate the regulation of target genes [26]. We therefore next explored the potential interaction between ERRγ and RUNX2 proteins by doing co-immunoprecipitation (Co-IP) of ERRγ and RUNX2 in MC3T3-E1 preosteoblast cell lines, transfected with either a full length ERRγ (ERRγ2), a LBD mutant (ERRγΔAF2), or a DBD mutant (ERRγC148G). All three ERRγ proteins were expressed, but only ERRγ2 and ERRγΔAF2 and not ERRγC148G co-immunoprecipitated with RUNX2 (Figure 6A), confirming the physical interaction of ERRγ and RUNX2 and revealing that the DBD is required for this interaction.

To determine whether RUNX2 is required for the *ERRγ* +/− osteoblast phenotype, we treated bone marrow stromal cells with antisense *Runx2* oligonucleotides post-confluence throughout the differentiation/mineralization period. *Runx2* AS but not S oligonucleotides significantly decreased RUNX2 expression (Figure 6B). Consistent with the observation that abrogation of *Runx2* expression has no overt effect on cells in cells at certain differentiation stages, i.e., in cells already committed to the osteoblast lineage [27], we observed no decrease in CFU-O or CFU-ALP in *Runx2* antisense-treated *ERRγ +/+* cultures treated in the time window tested (Figure 6C). In contrast, CFU-O and CFU-ALP were significantly decreased in *ERRγ +/−* cultures (Figure 6C). Taken together, the data suggest that the enhanced osteoblast differentiation seen in *ERRγ +/−* mice was both RUNX2-dependent and differentiation stage-dependent.

## Discussion

We report that whereas ERRγ-deficient mice manifested no detectable defects in embryonic or early postnatal development, ERRγ-deficient male mice exhibited increased trabecular, but not cortical bone volume in 14-week animals due to RUNX2-dependent negative regulation of the progression of osteoblast differentiation. Female mice were unaffected by ERRγ deficiency.

The increase in trabecular bone volume seen in *ERRγ +/−* male mice was due to increased trabecular number and thickness, as a consequence of an increased number of osteoblasts on the bone surface, increased BFR and increased MS/BS ratio, indicating an overall increase in active osteoblasts occupying the bone surface. We found no significant change in osteoclast number or surface, suggesting that ERRγ regulates bone formation and osteoblast differentiation, but not bone resorption. This was further supported by the observation of increased expression of certain osteoblast markers in the trabecular bone, with no change in markers of osteoclast formation or activity. Our findings contrast with ERα deficiency in male mice, which also leads to increased trabecular bone volume, but due to decreased bone turnover, with decreases in both bone formation and resorption [25]. On the other hand, ERRγ deficiency also results in increased trabecular bone volume, due to increased bone formation, resulting from increased osteoblast differentiation; this was variously reported to be accompanied by no differences in CFU number or proliferation in female mice only [9] or with an increase in osteoblast proliferation, with no increase in CFU number in both male and female mice; these discrepancies may reflect differences in mouse strains and/or the targeting strategies used [10]. In any case, we detected no significant changes by µCT analysis in *ERRγ +/−* female mice at the same ages at which differences were seen in *ERRγ +/−* versus *ERRγ +/+* male mice.

The fact that a bone consequence of ERRγ haploinsufficiency is seen only in adult male mice, and not in developing or neonatal mice or in female mice at any age tested is of interest. This is likely not due to age-related changes in expression levels of ERRγ, since we have found ERRγ to be expressed at relatively low levels in male and female bone (similar to ERα levels) at all ages tested, and expression is flat/constitutively low in cultures undergoing osteoblast differentiation (data not shown). It is possible that ERRγ has a redundant role in endochondral bone or growth plate development and bone growth, but we also observed no change in BV/TV in newborn mice, which suggests that ERRγ is not necessary for osteoblast differentiation or precursor fate at developmental or neonatal stages, but rather plays a regulatory role in mice only at sexual maturity. This suggests that negative regulation of osteoblast differentiation by ERRγ is sex hormone-dependent. Regulation of sex hormone receptor-dependent bone phenotypic outcomes is complex and multifaceted, but it is notable that sex-dependent adult bone phenotypes have also been seen in ER knockout mice. For example, ERα ablation decreased bone turnover and increased trabecular bone mass in both male and female mice, whereas ERβ ablation decreased bone resorption and increased trabecular bone mass only in female mice [25]. The elevated BV/TV observed in the male gonad-intact ER knockouts was found to be due to the high serum testosterone levels and androgen receptor (AR) function [25]. We therefore tested but found no difference in serum testosterone levels between *ERRγ +/−* and *ERRγ +/+* mice. While complete elucidation of the underlying direct and indirect mechanisms for sex-dependent effects on bone of ERs or AR in a background of altered ERRγ expression is ongoing, interactions between ER and ERR signaling pathways and transcriptional activity would not be unexpected to play roles in mice with altered expression of ERRs. Specifically with respect to ERRγ, estrogen via ERα has been shown to regulate ERRγ expression in breast cancer cells [28]. Transcriptional coactivators such as peroxisome proliferator-activated receptor γ (PPARγ) coactivator-1 α (PGC-1α) and PGC-1β amongst others, play important roles in both ER- and ERR-mediated transcription [29]–[32], suggesting that transcriptional cofactors are partially shared between ERs and ERRs, such that altered levels of ERRγ may alter cofactor stoichiometry for ERs and other ERRs. ERRs can bind to both estrogen response elements (EREs) and ERR response elements (ERREs) [2], [33]–[35], suggesting that ERRs can also affect ER-mediated signaling. Consistent with this, ERRγ has been shown to modulate ERα responsiveness in prostate, breast, and uterine endometrial cancers [28], [36], [37]. Thus, we postulate that ERRγ haploinsufficiency may alter the actions of ERα in male mouse bone, an effect potentially abrogated by ERβ activities and estrogen signaling in females. Of course, since ERRs can form not only homo- but heterodimers with each other and ERs [2], [34], [38]–[42], we cannot rule out ER-independent effects of ERRγ on bone target genes and bone or a role for ERRα or potentially ERRβ in the bone phenotype seen.

Our observations on bone marrow stromal cells *in vitro* indicate that ERRγ deficiency leads to an increase in osteoblast differentiation (increase in CFU-ALP and CFU-O), without a change in the number of mesenchymal precursors (CFU-F) or proliferation. Further, we observed an increase in *Alp* and *Bsp*, markers of osteoblast differentiation and activity. Although we cannot rule out the possibility that ERRγ directly regulates these genes, previously, Jeong *et al.* showed that ERRγ repressed the transcriptional activity of RUNX2 on *Bsp* and *Ocn* [15]; they also showed that ERRγ physically interacted with RUNX2, an interaction that repressed RUNX2 transactivity on a 6x OSE-luc promoter construct [15]. We now show that ERRγ interacts with RUNX2 *in vitro*, and that the DBD is necessary for this protein interaction, indicating that the DBD is responsible not only for binding to response elements, but also for proper protein-protein interaction. It has been reported previously that both the LBD and the DBD contain regions for dimerization [42]. Interestingly, it has been shown that ERα interacts with RUNX2 in MCF7 breast cancer cell line, and the MC3T3-E1 pre-osteoblast cell line, and that this interaction was estrogen dependant, and resulted in the inhibition of RUNX2 activity [43]. Further, these authors reported that the DBD harboured the interaction motif that directly binds RUNX2. Thus, one can hypothesize that a larger transcription complex, including ERRγ and ERα may form to modulate RUNX2 activity, and regulate osteoblast differentiation/mineralization. Our data suggest that ERRγ may bind to DNA while interacting with RUNX2. There is no reported putative ERRE within the 6x OSE response element, and it has not yet been shown that ERRγ can bind this region directly.

*Alp* and *Bsp*, but not *Runx2*, *Osx* or *Ocn* are upregulated in *ERRγ +/−* compared to *ERRγ* +/+ trabecular bone samples and in differentiating stromal cell cultures, suggesting that ERRγ negatively regulates osteoblast differentiation and matrix mineralization at a stage after osteoblast commitment (i.e., after upregulation of *Runx2* and *Osx* expression) but before osteoblast maturation or terminal differentiation to osteocytes (i.e., prior to acquisition of *Ocn* expression, and expression of osteocyte markers *Dmp1* and *Sost* [data not shown]). This, together with the observation that *Runx2* antisense treatment of post-confluent differentiating stromal cell cultures significantly reduced CFU-O and CFU-ALP in *ERRγ +/−* cultures compared to sense controls, but not in *ERRγ +/+* cultures, supports the conclusion not only that RUNX2 is required for the *ERRγ* +/− osteoblast phenotype but that ERRγ's role is differentiation stage-dependent. This is interesting in view of recent data that *Runx2* also has differentiation stage-dependent effects, with no effect of knockdown or knockout at certain developmental times. Thus, although RUNX2 was originally thought to be necessary at all stages of osteoblast differentiation, it has now been shown via analysis of the α*1(I)-collagen-Cre;Runx2^flox/flox^* (2.3 kb α*1(I)-collagen* promoter) conditional knockout mouse that *Runx2* does not have an overt effect in cells already committed to the osteoblast lineage [27]. On the other hand, several kinds of data, including results from the global *Runx2* knockout and an *OG2-ΔCbfa1* transgenic mouse indicate that RUNX2 is required in uncommitted osteoprogenitors [44] and in mature OCN-expressing osteoblasts [45]. The lack of effect of *Runx2* antisense knockdown in *ERRγ* +/+ cells is consistent with the fact that the majority of osteoblast precursors in the mouse cultures under the experimental conditions used are already committed and differentiating at the time of antisense treatment [46]. Further analysis of additional genetically-modified mouse models will be useful for extending the evidence that ERRγ is a differentiation stage-dependent negative regulator of osteoblast differentiation.

In summary, this is the first report of the consequences of ERRγ ablation and haploinsufficiency on bone metabolism *in vivo* and extends previous reports of ERRγ function in osteoblasts to the whole animal level. Our data indicate that ERRγ is a sex-dependent and RUNX2-dependent negative regulator of osteoblast differentiation, and that its regulatory activity may also be differentiation stage-dependent.
